# Bridging the gap: a review of dose investigations in paediatric investigation plans

**DOI:** 10.1111/bcp.12402

**Published:** 2014-09-19

**Authors:** Lisa V Hampson, Ralf Herold, Martin Posch, Julia Saperia, Anne Whitehead

**Affiliations:** 1 Medical and Pharmaceutical Statistics Research Unit, Department of, Mathematics and Statistics, Lancaster UniversityLancaster, UK; 2 European Medicines Agency7 Westferry Circus, London, UK; 3 Centre for Medical Statistics and Informatics, Medical University of ViennaSpitalgasse 23, Wien, Austria; 4 Medicines and Healthcare Products Regulatory Agency151 Buckingham Palace Road, London, UK

**Keywords:** Bayesian methods, dose investigations, extrapolation, modelling, simulation

## Abstract

**Aims:**

In the EU, development of new medicines for children should follow a prospectively agreed paediatric investigation plan (PIP). Finding the right dose for children is crucial but challenging due to the variability of pharmacokinetics across age groups and the limited sample sizes available. We examined strategies adopted in PIPs to support paediatric dosing recommendations to identify common assumptions underlying dose investigations and the attempts planned to verify them in children.

**Methods:**

We extracted data from 73 PIP opinions recently adopted by the Paediatric Committee of the European Medicines Agency. These opinions represented 79 medicinal development programmes and comprised a total of 97 dose investigation studies. We identified the design of these dose investigation studies, recorded the analyses planned and determined the criteria used to define target doses.

**Results:**

Most dose investigation studies are clinical trials (83 of 97) that evaluate a single dosing rule. Sample sizes used to investigate dose are highly variable across programmes, with smaller numbers used in younger children (< 2 years). Many studies (40 of 97) do not pre-specify a target dose criterion. Of those that do, most (33 of 57 studies) guide decisions using pharmacokinetic data alone.

**Conclusions:**

Common assumptions underlying dose investigation strategies include dose proportionality and similar exposure−response relationships in adults and children. Few development programmes pre-specify steps to verify assumptions in children. There is scope for the use of Bayesian methods as a framework for synthesizing existing information to quantify prior uncertainty about assumptions. This process can inform the design of optimal drug development strategies.

**What is already known about this subject**

- Paediatric dose investigations pose methodological and ethical challenges.
- Where appropriate, paediatric dosing recommendations should be informed by existing data.

**What this study adds**

- A variety of strategies are used to support paediatric dosing recommendations reflecting differing amounts of caution about dose.
- Pre-specified strategies for verifying extrapolation assumptions in children are often undefined.
- There is scope for using Bayesian methods to quantify prior knowledge when there is uncertainty about extrapolation assumptions.

## Introduction

Recent regulatory changes place greater emphasis on ensuring that new medicines are appropriately licensed for use in children. The EU Paediatric Regulation (EC 1901/2006) mandates the development of new medicinal products in children from birth to 18 years where there is a therapeutic need. Development should follow a prospectively agreed paediatric investigation plan (PIP). This plan must be submitted by an ‘applicant’ for the consideration of the Paediatric Committee (PDCO) of the European Medicines Agency (EMA) no later than completion of the adult pharmacokinetic (PK) studies; requests for a waiver of development must be justified. Most applicants are pharmaceutical companies.

Finding an appropriate dosing rule for a new medicine is crucial if its benefits are to be properly evaluated. Notwithstanding the ethical challenges, there are several methodological challenges peculiar to dose finding in children. Differences in the pharmacokinetics and pharmacodynamics of a drug across age groups, reflecting changes in developmental growth and maturation 1,2, may result in differences in possible clinical benefit. Relationships between body weight and pharmacokinetics may also vary with age 3. Information on drug pharmacokinetics and pharmacodynamics, therefore, is usually needed across all ages concerned by development to identify subgroups for which different optimal dosing rules apply. The ICH E11 guideline 4 proposes one possible age grouping, classifying children as neonates (0–27 days); infants (1–23 months); children (2–11 years) or adolescents (12–17 years), but cautions that unnecessary age subdivisions may unnecessarily increase the number of patients required.

The experimental effort needed to support dosing recommendations in children should be relative to the uncertainty that exists about likely drug effects in this population at the time a PIP is designed. If a medicine is first in class, that is, has a novel mechanism of action, or is intended to treat an exclusively paediatric condition, investigators may have little to guide them about the likely form of the dose−response or pharmacokinetic-pharmacodynamic (PK-PD) relationship. In such cases, dose finding trials in children should be designed accounting for this uncertainty, perhaps evaluating multiple dosing rules chosen to enable accurate model fitting and estimation under a variety of plausible models 5. In the case of dose escalation studies, escalation over a dosing-set that is usually limited in children compared with adults may be guided by accumulating PK or PD data to increase efficiency and safety 6. Acceptable designs for dose finding trials in children may be shaped by ethical considerations or constraints on feasible sample sizes, where the latter may apply when diseases are rare.

Fewer doses may be sufficient to establish the dose−efficacy relationship in children if one can extrapolate from data in a related population. In addition, PD or PK endpoints may be used as surrogates for efficacy. To illustrate this, suppose one can assume exposure−response (E−R) relationships are similar in adults and children. Then an optimal dosing strategy in children is found by targeting in every age group levels of exposure thought to be efficacious and safe in adults. This approach has been termed ‘complete extrapolation’ of efficacy data 7 and has been used successfully to support paediatric approval of the anti-epileptic drug oxcarbazepine 8. If relevant data are numerous, the need for a clinical trial in children may be obviated entirely, with dosing rules supported by modelling and simulation (M&S) alone. The value of M&S for optimizing paediatric drug development is recognized 9,10.

When data are extrapolated from a related population (referred to as the source population) to children (referred to as the target population), the quality of inferences about children depends both on the accuracy of the assumptions underpinning the extrapolation and the quality of the extrapolated data. Complete extrapolation of efficacy from adults to children is not possible if the adult E−R relationship is poorly characterized 11. An EMA concept paper 12 proposes using emerging data to corroborate assumptions as part of an overarching framework for extrapolation. We distinguish between verifying and validating assumptions, where the former implies generating supportive evidence and the latter definitive evidence.

We have undertaken a review to explore current strategies adopted in PIPs to support paediatric dose recommendations. Our aim was to record common assumptions underlying dose investigation strategies and the steps planned to verify them in the paediatric population. When study results have been reported, we can then evaluate the usefulness of various strategies for dose selection.

## Methods

A PIP application must detail the condition, indication(s) and age group concerned by development and outline all of the studies intended to support this. If an application proposes that the medicine treat more than one condition, a PIP must be specified for each condition targeted. If the PDCO judges a PIP to be acceptable, or can agree upon suitable modifications with the applicant, it adopts a positive PIP opinion. An opinion is a formal document summarizing the binding elements of the agreed development plan.

Information for this review was extracted from opinions and, where necessary, PIP applications and applicant responses to PDCO comments. Opinions are publicly available at http://www.ema.europa.eu/ema/index.jsp?curl=pages/medicines/landing/pip_search.jsp&mid=WC0b01ac058001d129. One author (LH) extracted the data seeking advice from others (RH, JS) on an *ad-hoc* basis. The protocol, data extraction form and pre-specified analysis plan for the review can be found in the supplementary material accompanying this manuscript. All statistical analyses were performed in R 13.

This review focused on clinical ‘dose investigation’ studies stipulated by PIP opinions. We adopted a broad definition of dose investigation study to include clinical trials that a) state a dose finding objective in the opinion or are cited as informing dose choices for subsequent trials or b) compare two or more dosing rules or c) measure PK end points. We included stand alone trials and portions of multistage trials. Furthermore, we regarded the following extrapolation approaches as dose investigating: a) PK, PK−PD or dose-PD M&S studies, b) prospective literature reviews gathering information on effective medicine use in children and c) use of completed paediatric dose investigation trials when listed in the opinion. Table 1 lists the terminology used in this paper.

**Table 1:** Glossary

Term	Definition
**Dose evaluation study**	Evaluates single dosing rule with the objective of verifying its pharmacological properties **Example:** See example A of the fixed dose strategy trial
**Dose finding study**	Evaluates multiple dosing rules to establish the dose-response relationship **Example:** See example B of the fixed dose strategy trial
**Dose investigation study**	General term encompassing dose evaluating and dose finding studies
**Dosing rule**	A rule specifying the amount of medicine to be given (and the administration schedule) in all age groups concerned by medicinal development. For example, if the dosing rule is x_1_ mg kg^−1^, different rules are defined by varying x_1_.
**Fixed dose strategy trial**	Trial evaluating a number of pre-specified dosing rules of a novel medicine. Trial may incorporate a control arm. Intra-individual dose adaptation permitted at a limited number of time points, with adjustments guided by PK/PD/efficacy responses **Example A:** Participants aged 1 month to less than 18 years receive novel drug according to the same regimen for 14 days. PK samples collected across day 14; safety and tolerability measured throughout. **Example B:** Double-blind, randomized, multicentre trial to compare the efficacy of two dose levels of a novel treatment with placebo and (one dose level of) active control. **Example C:** Initial doses for participants aged from 2 to less than 18 years set by weight categories. Treatment maintained for 2 years but PK measured within first 14 days of treatment. In-stream adjustment of individuals’ doses permitted if individual PK parameters deviate from predicted values based on adult PK data.
**Maximum tolerated dose (MTD)**	The highest dose associated with an acceptable risk of toxicity. The 3+3 dose escalation procedure 19, which treats patients in cohorts of three, finds the MTD as follows. Suppose doses {d_1_, d_2_, …, d_K_} are available for consideration. If two or more of the first three patients treated at a dose level d_i_ experience a dose limiting toxicity (DLT), the procedure stops declaring d_i-1_ as the MTD. Otherwise, if one of the three patients treated with d_i_ experiences a DLT, a further three patients are treated at this dose level. Then, if two or more of the six patients treated at dose level d_i_ experience a DLT, the procedure stops declaring dose d_i-1_ as the MTD. If continuation of the trial is permitted, the procedure escalates to treat the next cohort at dose d_i+1_.
**Non-randomized crossover trial**	Each patient is treated according to two different dosing rules which are administered in the same sequence to all patients. Total dose received need not escalate between successive dosing periods **Example:** All participants from birth to less than 18 years of age receive study medication twice daily for 4 weeks, followed by a 4 week treatment period of once daily dosing. In each treatment period, patients receive up to 2 mg kg^−1^ day^−1^ of study medication (so the dosing schedule varies over periods but total amount of drug received per day is held constant). Trial objective is to compare the pharmacokinetics of once daily dosing *vs*. twice daily dosing.
**Sequential cohort dose escalation trial**	Patients are treated in cohorts. Successive cohorts either escalate through a set of dosing rules (forced dose titration) or escalation decisions are determined by trial data **Example A:** Five cohorts of eight adolescents enrolled sequentially. Patients in a cohort receive the same dose. Doses received by cohorts 1 to 5 escalate from 0.5 mg to 5 mg, respectively. Patients treated for 14 days. (Fixed dose phase may be preceded by dose titration phase depending on maximum dose for the cohort.) **Example B:** Patients aged from 1 to less than 18 years enrolled in cohorts of three. Dose escalation proceeds on the basis of a 3 + 3 design 19, with dose recommendations for the next cohort determined by accumulated trial data, specifically dose limiting toxicities experienced by patients in their first cycle of therapy. Starting dose is 80% of adult MTD, with scaling based on body surface area.
**Target dose criterion**	Criterion defining dosing rule sought by the dose investigation study or the dosing rule that will be taken forward for use in subsequent trials (or to inform labelling).
**Therapeutic dose monitoring (TDM) trial**	Trial stipulating a flexible dosing strategy adapting to patients’ efficacy/PD/PK response, with no fixed dosing rule envisaged **Example:** In children aged 2 to less than 18 years, starting daily doses will be body surface area equivalent of 6g day^−1^ in adults. Thereafter, study medication is to be titrated up and down as needed following a developed scheme to maintain age appropriate levels of an efficacy endpoint. Trial objective is to determine the initial starting doses of study medicine for children.
**Within-subject dose escalation trial**	Each patient is treated according to multiple dosing rules. Successive doses either escalate through a pre-specified set (forced dose titration) or escalation decisions are determined by trial data. **Example:** Patients aged 8 to less than 18 years initiated on 2.5 mg of study medication. Dosing then doubled at 4 weekly intervals, with escalation decisions determined by accumulating safety and tolerability data. PK assessments made throughout period of dose escalation. Treatment at an individual's MTD is maintained until week 24.

We excluded from our definition of dose investigation study a) pre-clinical studies (including *ex vivo* studies), b) bioequivalence trials, c) therapeutic dose monitoring (TDM) trials without a stated dose finding objective and d) efficacy bridging studies not making explicit mention of pharmacological modelling.

From each PIP, information was extracted about all studies classified as dose investigating according to our stated criteria. If a PIP stipulated two dose investigation studies to determine dosing rules for the medicine when administered as monotherapy and adjunctive therapy, we extracted information for both.

If an opinion covered more than one condition, we extracted information for each unique PIP specified. If there was some overlap between the clinical studies comprising PIPs for different conditions, we recorded for each PIP only the most relevant dose investigation study(s), where ‘most relevant’ meant that studies conducted in a much broader population were not counted. For example, suppose a PIP application targeted two conditions: *solid tumours excluding melanoma* and *melanoma*. If a dose escalation study was planned in patients with all types of solid tumour, along with a M&S study to establish dose in melanoma patients, we would record the first study as dose investigating for *solid tumours excluding melanoma* but only the second study as dose investigating for *melanoma*. This type of scenario concerned three PIP applications. Henceforth, we will refer to PIPs as ‘development programmes’.

We hypothesized that rare diseases are a trigger for extrapolation as applicants seek to augment small sample sizes. For a medicine to be recommended for an orphan designation by the EMA Committee for Orphan Medicinal Products, amongst other criteria the treated condition must not affect more than 5 in 10 000 people in the EU 14. We used a medicine's orphan status as a surrogate for the prevalence of the condition to be treated. However, an applicant is at liberty to seek an orphan designation for its product at any point during development before marketing authorization. We accorded a medicine orphan status if designations had previously been recommended for other medicines treating at least one of the conditions or indications targeted by the PIP. This information was extracted from the EMA online database of orphan recommendations 15 on 27 February 2013.

## Results

We extracted information from the last 74 opinions agreeing a PIP that had been adopted by the PDCO as of 20 July 2012, spanning a period of about 14 months, excluding opinions granting a full development waiver. Of the opinions reviewed, one was excluded because dose investigations were judged unnecessary by the authors of this manuscript. The review focused on the remaining 73 opinions, representing 79 ‘development programmes’ spanning 17 therapeutic areas (TAs; see Table 2). Of these, 29 programmes concerned orphan medicines and 14 concerned medicines already holding a marketing authorization in the EU. Sixty-eight (of 79) programmes planned at least one dose investigation study and 59 (of 79) planned dose investigations in every age group for whom use of the medicine was considered relevant by the PDCO. Sixty-eight programmes investigating dose planned a total of 97 dose investigation studies.

**Table 2:** Descriptive statistics for the 79 development programmes. Data are listed as number (%)

Therapeutic area†	
**Oncology/haematology-haemostaseology**	23 (29.1%)
**Pneumology-allergology/infectious diseases**	11 (13.9%)
**Immunology-rheumatology-transplantation**	10 (12.7%)
**Psychiatry/neurology**	9 (11.4%)
**Cardiovascular diseases**	6 (7.6%)
**Vaccines**	5 (6.3%)
**Endocrinology-gynaecology-fertility-metabolism**	5 (6.3%)
Other*	16 (20.3%)

**Regulatory status of the medicine**	
**Medicines that are not yet authorized in the EU**‡	65 (82.3%)
**Medicines already authorized in the EU**	14 (17.7%)

**EU orphan designation**	
**Yes**	29 (36.7%)
**No**	50 (63.3%)

**Year of submission of PIP application**	
**2010**	8 (10.1%)
**2011**	70 (88.6%)
**2012**	1 (1.3%)

† Percentages sum to > 100% as the condition targeted by a PIP may cover more than one therapeutic area.

* The category ‘Other’ covers the following therapeutic areas: gastroenterology-hepatology, diagnostic, dermatology, ophthalmology, neonatology-paediatric intensive care, uro-nephrology and ‘other’.

‡ Including medicines where the patent has expired and the development of the product in children is voluntary.

### Design of dose investigation studies

Table 3 classifies dose investigation studies according to their design. Supplementary Tables 1 and 2 summarize the 83 dose investigation studies that were clinical trials according to the data sources used to determine starting doses and measured end points. One type of dose investigation study is an extrapolation exercise (as defined in the Methods section above). Programmes using extrapolation to investigate dose covered the following TAs: dermatology, endocrinology-gynaecology-fertility-metabolism, haematology-haemostaseology, immunology-rheumatology-transplantation, oncology, psychiatry and vaccines.

**Table 3:** Designs of 97 dose investigation studies planned by 79 paediatric development programmes. Listed are the median and interquartile range (IQR) of the median ages to be recruited in the different studies (or the median ages study conclusions apply to in the case of extrapolation exercises)

Dose investigation study design	Number of studies (by rarity of disease)		Median age (years) (IQR)	Median number of patients per study (IQR)
	Orphan	Non-orphan		
**Sequential cohort dose escalation trial (with intra-subject escalation)**	2 (5.0%)	1 (1.8%)	15 (2.8)	30 (18)
**Sequential cohort dose escalation trial (without intra-subject escalation)**	6 (15.0%)	5 (8.8%)	9.3 (0.5)	40 (24.5)
**Within subject dose escalation trial**	4 (10.0%)	4 (7.0%)	11 (2.9)	22 (15)
**Non-randomized cross-over trial**	1 (2.5%)	0	9 (0)	15 (0)
**Fixed dose strategy trial (> 1 dosing rule of novel medicine)**	5 (12.5%)	13 (22.8%)	11.3 (5.1)	61.5 (93)
**Fixed dose strategy trial (1 dosing rule of novel medicine)**	15 (37.5%)	25 (43.9%)	9.6 (2.6)	30 (18.5)
**TDM trial**	1 (2.5%)	1 (1.8%)	8 (2)	47.5 (2.5)
**Extrapolation exercise:**	6 (15.0%)	8 (14.0%)	9.3 (4.6)	0
**• M&S study**	2	5		
**• Prospective literature review**	2	1		
**• Citation in PIP of completed paediatric dose investigation trial in current medicine**	2	1		
**• Citation in PIP of completed paediatric dose investigation trial in related medicine**	0	1		

Many dose investigation studies (40/97) were dose evaluating in nature rather than dose finding. For this approach to be pertinent there should be a high degree of confidence in the proposed dosing rule. Otherwise, if it is found to be inadequate, the findings of a single arm PK study can be extrapolated to inform selection of an alternative dosing rule only under strong assumptions, i.e. assumptions that have far reaching clinical consequences yet are supported by limited evidence, in this case dose proportionality. Under this assumption exposure, i.e. area under the curve (AUC), is a constant multiple of dose; see Section 7.3 of 16. However, this cannot be verified using data from a single arm study. Twelve (of 40) of the fixed dose evaluating trials planned an interim analysis for dose confirmation, reflecting a degree of caution about dose. In Table 3, these 12 trials are classified according to the minimum number of dosing-rules evaluated.

Of the 18 fixed dose strategy trials evaluating two or more dosing rules, 14 and four trials planned to compare two and three rules, respectively. Comparing few dosing rules limits the complexity of any dose−response model that can be fitted: if two rules are compared, a linear model is the most complex description of the data possible.

Adaptive designs provide investigators with the flexibility to respond to emerging trial data and M&S is a useful tool to guide adaptations. Of the 23 fixed dose strategy trials that planned to stagger recruitment into vulnerable subgroups, 13 will use accumulating data to modify starting doses for later groups. Of these 13 trials, nine will use M&S to guide dose changes. However, few dose escalation trials planned to incorporate M&S into decision making. Instead almost all (21/22) will use algorithmic rules, unspecified safety analyses or unspecified PK analyses to guide escalation decisions.

Efficacy trials give an opportunity for proposed dosing rules to be assessed against clinical outcomes. A high proportion of dose investigating programmes (50/68) included trials labelled (in the opinion) as investigating efficacy, activity or immunogenicity in every age group concerned by development. This proportion drops in programmes using extrapolation (5/14).

### Sample sizes supporting dose recommendations

**Figure 1:**
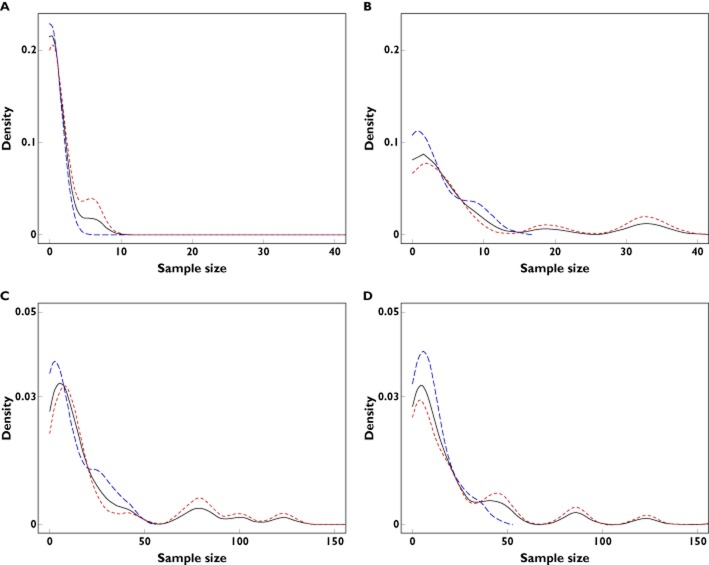
Densities of cumulative sample sizes accrued across dose investigation studies planned by programmes developing in an age group and stratifying recruitment. Listed are numbers of programmes developing in each age group. The sample size range is [0−40] in the top panel and [0−150] in the bottom. Overall median samples per age group are: 0.1 aged 0–27 days; 2.9 aged 1–23 months; 10.0 aged 2–11 years; 9.0 aged 12–17 years. 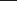, overall; 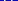, orphan; 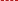, non-orphan(A) 0–27 days; 15 programmes. (B) 1–23 months; 23 programmes, (C) 2–11 years; 41 programmes and (D) 12–17 years; 47 programmes

Opinions stipulate total trial sample sizes, often stratified by age cohort to ensure there is a good reflection of the target population in the trial. We extracted this information to investigate whether the experimental effort used to support dose recommendations varies with age. We excluded from our analysis 19 (of 68) programmes that included one or more dose investigation clinical trial not providing information on the distribution of patients across age groups. Figure 1 plots kernel density estimates for cumulative sample sizes planned across dose investigation studies included in the remaining 49 (of 68) programmes. For this analysis, extrapolation exercises were recorded as recruiting 0 patients in every age group concerned by the exercise. For all other studies, sample sizes were broken down according to the ICH E11 age classification. If age cohorts stipulated by opinions did not coincide with the ICH E11 groupings, sample sizes for these groups were calculated assuming that within each cohort that was stipulated, recruitment would be evenly spread across the age range. Only programmes developing a medicine for use in the stated ICH E11 age group (as stipulated by the PIP opinion) were included in this sample size analysis.

For 11 trials within the 49 programmes, opinions stipulated minimum sample sizes per age cohort (summing to less than the required total sample size). In these cases we assumed that the sample size at the applicant's discretion would be recruited from the oldest age group under development. This tacitly assumes that older children are more readily recruited. The results of Figure 1, therefore, represent a worst case analysis.

There is a clear discrepancy between the sample sizes used to investigate dose in younger (<2 years) and older (>2 years) children. We infer that it is common to extrapolate evidence across age groups to support dose recommendations in the youngest children. Sample size distributions in older children are multimodal because a number of programmes planned large dose-ranging trials in these groups. These programmes covered the TAs pneumology-allergology, psychiatry, neurology and uro-nephrology.

**Figure 2:**
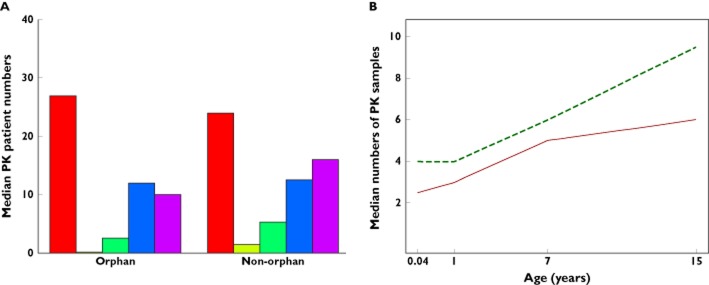
(A) Cumulative numbers of patients contributing PK information and (B) median numbers of PK samples per patient in 25 programmes conducting at least one PK trial, stratifying randomization into PK studies by age and stipulating intended sampling schedules. Numbers in each age group are based on programmes developing in that age group. Three (of 25) programmes obtain (some) PK samples from urine or saliva.(A) Cumulative PK patient numbers. 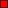, total; 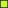, 0–27 days; 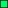, 1–23 months; 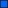, 2–11 years; 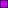, 12–17 years and (B) median numbers of PK samples per patient. 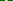, non-orphan; 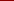, orphan

When PK parameters are measured, the precision of the parameter estimates will depend on sample size and sampling intensity, where the latter may also vary with age. We defined a PK study as a trial declaring PK as either a primary or secondary end point. Figure 2A displays the cumulative numbers of patients participating in PK studies for the 25 (of 68) programmes that included PK studies stratifying recruitment by age and stipulating a sampling schedule. Again, there is a discrepancy between the numbers of the youngest and oldest children participating in PK studies. Figure 2B shows that there is a strong positive association between age and the median number of PK samples per patient.

### Analysis of dose investigation studies

Modelling provides a more informative summary of the data from a dose investigation trial than descriptive statistics alone could give 17. Table 4 summarizes the methods of analysis cited by 81 dose investigation trials measuring at least one efficacy, PD or PK end point. PK modelling is widely applied, although complex physiologically based models are seldom fitted. For 23 trials, paediatric data will be pooled with adult data or observations from other paediatric trials to obtain greater precision for model parameter estimates.

**Table 4:** Techniques to be used to analyze 81 dose investigation studies that were clinical trials measuring at least one efficacy, PD or PK end point

Analysis technique	
**Descriptive analyses** ***summary statistics including confidence intervals; graphics; summaries of PK or PD parameters***	73 (90.1%)
**PK modelling** ***fixed effect or population PK models***	41 (50.6%)
**PK**–**PD modelling** ***including exposure***–***response, PK***–***response models***	17 (21.0%)
**Dose**–**response modelling** ***including dose-PD (e.g. ANCOVA model), dose***–***toxicity, dose***–***PK***–***PD models***	10 (12.3%)
**Physiologically-based PK modelling**	3 (3.7%)
**Dose**–**exposure modelling**	3 (3.7%)
**Other** ***Formal hypothesis testing on efficacy or PD end points; non-parametric time-to-event analyses; other types of models not captured above***	22 (27.2%)

### Target dose criterion

Table 5 lists the target dose criterion stated for 97 dose investigation studies. PK data alone will be used in 33 studies to guide dose selection with most (25/33) determining dosing rules by targeting levels of exposure seen in older subjects. Few of the 33 studies that were clinical trials pre-specified steps for verifying an assumption of similar E-R relationships in a source population and children: 20 (of 29) trials will measure an efficacy or PD endpoint and six of these will model the E−R relationship.

**Table 5:** Target criteria to be used by dose investigation studies conducted by 79 paediatric development programmes. Data from a total of 97 studies were extracted

Dose criteria	Number of studies (by rarity of disease)		
	Overall	Orphan	Non-orphan
**PK guided**	33 (34.0%)	9 (22.5%)	24 (42.1%)
**PK and PD/efficacy guided**	10 (10.3%)	3 (7.5%)	7 (12.3%)
**Maximum tolerated dose**	7 (7.2%)	6 (15.0%)	1 (1.8%)
**Optimum biologic dose**†	4 (4.1%)	1 (2.5%)	3 (5.3%)
**Other**	3 (3.1%)	1 (2.5%)	2 (3.5%)
**Not known**	40 (41.2%)	20 (50.0%)	20 (35.1%)

† Optimum biologic dose is one defined on the basis of PD or efficacy end points.

Other stated target dose criteria were vague. The maximum tolerated dose (MTD) 18, as determined by algorithmic dose escalation procedures such as the 3 + 3 design 19, is not well defined in the sense that it does not correspond to a fixed probability of a dose limiting toxicity (DLT). Indeed, the 3 + 3 design and close variations on it may terminate with an MTD associated with a DLT risk of 10–29% 20. A target dose criterion could not be determined for a high proportion of studies, particularly for those studies developing orphan medicines. By not pre-specifying a target criterion, applicants are flexible to respond to trial data although it is unknown whether a particular study design will be adequate to address the ultimate dose finding objective.

## Discussion

Few children less than 2 years of age contribute to dose investigations, suggesting that this age group is currently under researched. This is in conflict with recent FDA guidance that paediatric PK studies should have adequate precision for estimating relevant parameters in every age group under consideration 21. Figure 1 provides evidence to support our initial hypothesis that sample sizes are related to the prevalence of the condition treated and anticipated recruitment difficulties. However, extrapolating to children with rare diseases can be challenging due to the limited amount of adult data available. Alternatively, rare disease trials in children may recruit across similar conditions to increase the sample numbers available, an accepted practice in early phase oncology trials.

Bayesian methods 22 were rarely cited to support development programmes included in this review. Of the dose investigation studies included, one cited Bayesian methods for study analysis and one to guide mid-trial dosing adaptations. In the Bayesian paradigm, before a trial is conducted prior probability distributions for unknown parameters are determined from expert opinion or historical data. Once new data become available, priors are updated to form posterior distributions. The advantage of using Bayesian designs for paediatric trials is that they provide a framework through which prior knowledge can be quantified and uncertainty, for example, about the size of differences between the source and target populations of an extrapolation, can be communicated. This ‘extrapolation concept’, adopting the terminology of the EMA concept paper on extrapolation 12, may be used to inform selection of an appropriate extrapolation strategy, although selection will also be informed by the feasibility and ethics of generating additional data in the target population. The EMA concept paper on extrapolation 12 proposes that ‘studies should focus on those complementary areas, e.g. age subsets, where the largest differences to the source population are expected’. We refer to this as ‘stochastic extrapolation’.

Numerous Bayesian approaches have been proposed in the statistical literature for the design of paediatric 23,24 and rare disease trials 25–27. With the Bayesian approach there is a trade-off between the gain in precision of estimates and the dependence of posterior inferences on the prior distribution. There is caution amongst statisticians and regulators about the use of informative priors to interpret the results of clinical trials 17, although we contend that the influence of an informative prior should not be a concern if the prior is well-founded. For this to be true, prior distributions for parameters in children should be determined by a rigorous assessment of what is already known, including pre-clinical data and knowledge of class effects when determinants of the pharmacokinetics and pharmacodynamics of a class of drugs, and the effect of age, are well understood. In the UK public health sector, funding applications for clinical trials must be supported by a systematic review of existing literature 28. A similar guideline could be stipulated for PIP applications to ensure that there is an objective framework within which existing sources of related data can be identified and their relevance assessed. Quantifying the current state of knowledge will help to inform dose selection decisions when evidence generation in the target population is deemed unethical or definitive sample sizes are not feasible, as may be the case, for example, when diseases are rare.

The paediatric decision tree (see Section 2.4 of 4) does not accommodate uncertainty about extrapolation assumptions. If an assumption is deemed reasonable, applicants proceed by treating it as fact. We refer to this as ‘deterministic extrapolation’. In this framework, strong assumptions remain unverified because they preclude from being collected those data that could reveal inconsistencies between the assumptions and the true state of nature. A stochastic approach to verifying assumptions allows updating of the measures of uncertainty about dose−response.

One limitation of this review is that data were extracted by a single author, although this was not a major concern because opinions are highly structured. The review also concerns a small subset of the more than 600 PIP opinions to have been adopted by the PDCO since 2007. A comprehensive review of all PIPs was deemed infeasible. Therefore we chose to restrict attention to a contemporary subset. However, further research could examine changes in dose investigating strategies since 2007. While beyond the scope of the current paper, it would also be of interest to follow up those PIPs which were surveyed to determine which data were ultimately most informative for devising an appropriate dosing regimen.

The 79 development programmes included should not be interpreted as independent research plans. There will be orthodoxies for investigating dose in certain diseases that all PIPs will follow, driven by regulatory guidelines and precedents established by the PDCO. Adopted dose investigation strategies may reflect a compromise between the strategies of the applicant and those of the PDCO. This review excluded pre-clinical studies, which in relevant model systems may contribute towards an understanding of the possible differences between adults and children. However, they may be more relevant for safety questions. This review has noted only what steps are pre-specified for verifying assumptions directly in the paediatric population. The data collected by this review do not allow us to comment on whether additional studies should have been specified for verification of assumptions.

The conclusions of this review presume that the information provided in PIP applications and opinions gives an accurate picture of how studies were designed and will proceed. If relevant information was omitted from applications this could bias our results. Specifically, applicants may eventually take steps to verify extrapolation assumptions in children, such as PK−PD modelling, which were not pre-specified in the PIP.

## Conclusion

A variety of strategies are used to investigate dose in children, with small sample sizes used in children less than 2 years old. Common assumptions underlying dose investigation strategies include dose proportionality and similar E−R relationships in children and a source population. There is scope for the increased use of pharmacological modelling as a tool for verifying extrapolation assumptions. Future research would develop a framework for prospectively determining what studies are needed in children given existing data in adults and other populations.

## Disclaimer

The views expressed in this article are the personal views of the author(s) and may not be understood or quoted as being made on behalf of or reflecting the position of the EMA or one of its committees or working parties.

The authors wish to thank the EMA Paediatric Committee for helpful discussions and Dr Thomas Jaki for comments on an earlier draft of the manuscript. The authors also thank two anonymous referees and the Executive Editor for comments which improved the clarity of the manuscript.

## Supporting Information

Appendix S1 Supplementary tables Appendix S2 Data extraction protocol Appendix S3 Statistical analysis plan Appendix S4 Data extraction form
