# Supplementary material for: Bridging the gap: a review of dose investigations in paediatric investigation plans
Source: Br J Clin Pharmacol. 2014 Sep 19;78(4):898–907. doi: 10.1111/bcp.12402 (PMC4239983; doi:10.1111/bcp.12402)
Supplement: Supplementary file 1 — Appendix S1 Supplementary tables Appendix S2 Data extraction protocol Appendix S3 Statistical analysis plan Appendix S4 Data extraction form [file bcp0078-0898-sd1.doc]

Supplementary material to the manuscript “Bridging the gap: A review of dose-investigations in paediatric investigation plans” by Hampson LV, Herold R, Posch M, Saperia J and Whitehead A.

**Appendix A:** Supplementary Tables

| Variable | Primary endpoint | Secondary endpoint |
| --- | --- | --- |
| Efficacy† | 26 (31.3%) | 49 (59.0%) |
| Pharmacokinetic | 44 (53.0%) | 29 (34.9%) |
| Pharmacodynamic | 7 (8.4%) | 23 (27.7%) |
| Safety‡ | 26 (31.3%) | 60 (72.3%) |
| Other* | 3 (3.6%) | 24 (28.9%) |
| Not known | 0 (0%) | 7 (8.4%) |

**Supplementary Table 1:** Primary and secondary endpoints to be measured by 83 dose-investigation studies that will be clinical trials. Percentages sum to more than 100% as trials may list several variables as primary and secondary endpoints.

† Includes endpoints listed as efficacy, (anti-viral) activity, exploratory, quality of life and immunogenicity (where appropriate).

‡ Includes endpoints listed as adverse events, tolerability, maximum tolerated dose.

* Includes endpoints listed as palatability, acceptability, pharmacogenetic factors, measures of doses used.

| Information source | Frequency cited |
| --- | --- |
| Adults | 65 (78.3%) |
| Older children | 7 (8.4%) |
| Children with related condition (treated with medicine of interest) | 5 (6.0%) |
| Children treated with different formulation of the medicine of interest | 5 (6.0%) |
| Children in correct age group, condition, treated with the relevant formulation of the medicine of interest | 19 (22.9%) |
| Children treated with related therapies | 12 (14.5%) |
| Other | 5 (6.0%) |
| Not known | 3 (3.6%) |

**Supplementary Table 2:** Sources of information cited to support starting dose choices for the medicinal product being developed in 83 dose-investigation studies that will be clinical trials. Percentages sum to more than 100% as several data sources may be cited to justify starting dose choices.

Supplementary Table 2 records the sources of information used to support starting dose choices in clinical trials before the trial commences (when recruitment into more vulnerable subgroups is staggered, we do not capture the sources of information used to determine dose modifications). We see from Table 2 that most paediatric trials use information from adults to determine starting doses in children. Less than half of trials (34/83) use M&S to support these dose choices.

**Appendix B:** Data Extraction Protocol

**Objectives:** The primary objectives of this methodological review are to establish what studies are undertaken in PIPs to inform the final dose choice (listed in any Marketing Authorisation application) for medicines for children. We also aim to:

- Explore the role of extrapolation in determining the information and criteria used to inform dose-selection and record the steps taken to validate dose choices.
- Make suggestions for the improving the design and conduct of dose-investigation studies in children.

**Data sources:** Information for this review will be extracted from binding PIP opinions (adopted by the Paediatric Committee) and, where necessary, initial PIP applications (submitted by applicants) and modified PIP applications in response to PDCO comments.

Each PIP must state the condition(s) the medicine is intended to treat, and is classified according to its therapeutic area (TA). If a PIP opinion stipulates development of a product in several conditions, we will create a separate record for each condition unless the *development programmes (*defined here as sequences of studies*)* are completely overlapping, in which case the PIP will be counted only once. Alternatively, if there is only some overlap in development between the conditions, we will record for each condition only the most relevant dose-investigation study(s). We will not break down studies planned by PIPs by patient subgroup. For example, if a PIP opinion for a HIV treatment stipulates different dose-investigation studies in treatment-naïve and -experienced patients, we will record this programme as planning 2 dose-investigation studies.

When discussing dosing requirements for paediatric medicines, if dose is normalised by weight or age group, the amount of medicine received will vary between individuals. In such cases, we seek the optimal ‘dose strategy’ rather than a single dose: for example, in the simplest case that dose is x_1_ mg/kg, different strategies are defined by varying x_1_.

**Definition of dose-investigation study:** We exclude from our definition of a dose-investigation study the following types of study:

1. Pre-clinical studies (in vivo/ex-vivo/animal studies)
2. Bioavailability, bioequivalence and systemic exposure studies comparing a new therapy with an accepted dose of a related therapy
3. Therapeutic dose monitoring (TDM) studies which do not have a stated objective of informing future starting dose choices.

We include all other clinical trials that:

1. State a dose-finding objective in the PIP opinion, or
2. Compare multiple dosing rules of the novel medicine, or
3. Measure pharmacokinetic endpoints.

We include PK and PK/PD studies sampling drug concentration-time profiles generated by a single dosing rule since such trials can inform dose selection if one is prepared to make strong assumptions, such as dose-proportionality. We include stand-alone trials and trials that are the first stages of seamless studies that go on to confirm dose-selection(s) by activity/efficacy investigations. More than one trial in a development programme may inform dose-investigations: we will extract information for each.

We include in our definition of dose-investigation study extrapolation based, or ‘non-interventional’, studies including:

1. PK or PK/PD, dose-PD modelling and simulation (M&S) studies,
2. Prospective literature reviews gathering information on effective drug use in children,
3. Citations of completed paediatric trials in the PIP opinion to support dose choices.

We exclude efficacy bridging studies not making explicit mention of PK or PK/PD modelling.

We detail below our approach to data extraction in order to meet the review objectives:

**Target dose criteria:** We will extract from positive PIP opinions details of the evidence generated by dose-investigation studies. Primary and secondary endpoints measured by dose-investigation studies that are clinical trials will be classified as: efficacy; pharmacokinetic; pharmacodynamic; or safety. Where possible, endpoints will be grouped according to the classification used in the PIP opinion.

We will record the criterion by which target doses for studies are defined. In most cases, this information will need to be extracted from the PIP application. Target dose criteria will be categorised as: maximum tolerated dose; optimum biological dose; PK guided dose selection; other; not stated.

**Use of extrapolation:** To explore the role of extrapolation in dose-investigations, we will record the frequency with which non-interventional studies are planned. We will investigate whether the frequency of non-interventional studies differs between PIP applications submitted under Articles 7 and 8 of the Paediatric Regulation, and those concerning medicines with and without an orphan designation.

If dose investigating clinical trials are planned, starting doses may be chosen on the basis of extrapolated information. We will record the frequency with which starting dose choices are informed by data generated in adults, older children, children with a related condition, children treated with a different formulation of the same medicine and children treated with a related therapy. Likely sources of extrapolated evidence are informed by the findings of Dunne et al. (Dunne J, Rodriguez WJ, Murphy MD et al. Extrapolation of adult data and other data in pediatric drug-development programs. *Pediatrics* 2011;128(5):e1242 - 1249).

**Aspects of design for paediatric dose investigation studies that are clinical trials:** We will classify dose-investigation clinical trials according to their design. Trials will be classified as: fixed dose-strategy trials; therapeutic dose-monitoring trials; within subject dose-escalation trials; sequential cohort dose-escalation trials without intra-subject escalation; sequential cohort dose-escalation trials with intra-subject escalation. We will record the age-range each trial is to be conducted in since the acceptability of different designs may depend upon the study population. To assess whether planned dose-investigation trials will generate sufficient evidence to estimate the underlying dose-response relationship, we will record the number of dosing rules that trials will compare.

Model-based dose-escalation procedures are typically more accurate or efficient than algorithmic designs. With this in mind, we will categorise rules used to make go/no-go dose escalation decisions as: forced titration; model-based (e.g., continual reassessment method, escalation with over-dose control); algorithmic (whether based on safety or pharmacological data). Rules for determining dose-increments when escalations are permitted will be recorded and classified as: algorithmic, targeting a certain level of response, or optimal design criterion. Under an optimal design “variance gain criterion”, the next patient(s) would be allocated the dose(s) that would reduce by the greatest margin uncertainty about the target dose: this criterion maximises statistical information but may be unethical in practice. For dose-investigation studies that we classify as fixed dose-strategy trials, we will record whether sponsors plan to make mid-study adjustments to doses if accumulating data suggest starting doses are sub-therapeutic.

To avoid exposing vulnerable patients to potentially toxic or sub-therapeutic doses, enrolment of children in dose-investigation clinical trials may be staggered. We will record whether recruitment into dose-investigation trials will be staggered, the criteria used to define paediatric subgroups and group sizes. Group sizes will likely reflect a trade-off between the aim of rapid trial completion and the need to verify drug safety or model accuracy before beginning testing in younger/lighter children.

**Appendix C:** Statistical Analysis Plan

We detail below the statistical analyses that will be conducted to address the key objectives of this review. All analyses will be descriptive in nature. No formal hypothesis testing is planned due to small sample sizes meaning that there is low power to detect deviations from the null. In the following, DE Q1 refers, for example, to Data Extraction Form Question 1; the final data extraction form is given in Appendix D.

# Analysis Sets

**Full Analysis Set:** Information will be extracted from the last 74 PIPs for active substances submitted to and adopted by the PDCO as of 20 July, 2012, excluding those applications for which a full waiver was granted. Of these, 1 PIP will be excluded for the reason that dose-investigations are not needed (as considered by the authors of this review). The development programmes represented by the remaining 73 PIPs will form the Full Analysis Set.

**Dose-Investigation Set (Programmes conducting dose-investigation studies):** This analysis set will include all development programmes planning clinical trials and/or extrapolation based exercises fulfilling our definition of a dose-investigation study.

We define 3 further analysis sets based on the Dose-Investigation Set:

**All Studies:** The set of all dose-investigation studies (clinical trials and extrapolation based exercises) planned by programmes in the Dose-Investigation Set.

**All Clinical Trials:** The set of all clinical trials planned by programmes in the Dose-Investigation Set.

**Dose-Varying Clinical Trials:** The subset of trials in the All Clinical Trials Set which plan to assess more than one dosing rule of the novel medicine.

Most analyses will be based upon the All Studies Analysis Set since this captures all current approaches used to inform dose recommendations in children.

# Summary of the Full Analysis Set

**Question 1: What therapeutic areas (TAs) are covered by programmes in the Full Analysis Set?**

**Using DE Q3:** Summarise the percentage of programmes classified according to each TA. TAs with two or fewer programmes will be combined.

**Question 2: How many programmes will conduct dose-investigation studies in all ages concerned by development?**

**Using DE Q6 - 8, Full Analysis Set:** Summarise the number of programmes that plan to conduct dose-investigation studies recruiting children spanning the entire age range for which use of the medicine is considered relevant by the PDCO.

# The role of extrapolation in dose-investigations

**Question 3:** **What information is dose-selection based upon?**

**Using DE Q10 - 11; All Clinical Trials:** Summarise the percentage of trials with a primary endpoint classified as: efficacy; PK; PD; or safety. Clinical trials with co-primary endpoints will contribute more than once to this analysis.

**Question 4: What criteria are used to define target doses?**

**Using DE Q19, All Studies (results broken down overall and by medicine orphan status):** Summarise the percentage of studies using the following criteria to define the target dose: maximum tolerated dose; optimum biologic dose; PK guided selection; PK & response guided selection (where response is PD and/or efficacy); other; not known.

**Question 5: What types of clinical trial design are used for dose-investigations in children? How often are extrapolation-based approaches used to support dose selection?**

**Using DE Q12 – 16, All Studies (results broken down overall and by medicine orphan status):** Summarise the percentage of planned dose-investigation studies that are classified by the authors of this review as:

- Fixed dose-strategy studies (assessing 1 dosing rule of novel medicine)
- Fixed dose-ranging studies (assessing >1 dosing rule of novel medicine)
- Therapeutic dose monitoring trials (targeting PK or PD response)
- Cross-over trials (comparing > 1 dosing rule of the novel medicine within patients)
- Within subject dose-escalation trials
- Sequential cohort dose-escalation trials without intra-subject escalation
- Sequential cohort dose-escalation trials with intra-subject escalation
- Non-interventional studies that synthesize existing data. Specifically:
- M&S PK, PK/PD/efficacy, dose-PD studies that extrapolate from existing data
- Prospective literature reviews
- Citations of completed dose-investigation trials conducted in adults and/or children in the novel medicine (if such completed studies are listed in PIP opinion)
- Citations of completed dose-investigation trials conducted in adults and/or children in a related medicine (if such completed studies are listed in PIP opinion).

**Question 6: Is the use of extrapolation to inform dose-selection determined only by the availability of existing evidence?**

**Using DE Q4 and Q5, Dose-Investigation Set:** Summarise the percentage of programmes submitted under Articles 7 or 8 of the Paediatric Regulation. Compare percentages with the frequency with which extrapolation based dose-investigation studies are planned.

**Question 7: If dose-selection is informed by clinical trial data, do investigators borrow strength from data collected in older children to inform dose recommendations in younger children?**

**Using DE Q12.4, Q13.4, Q14.4, Q15.4 Q16, Programmes planning at least 1 dose-investigation study providing information on the distribution of patients across age groups. Results broken down overall and by orphan status:** Pooling across all dose-investigation studies planned by a development programme, summarise the number of patients planned in 4 age groups (as defined in the ICH E11 guidance document). Exclude from this analysis programmes that include trials planning to recruit across the ICH E11 age groups without stipulating how the total sample size would be broken down (because in such cases, we have no reasonable means of determining how the total sample size will be spread across the ICH E11 age groups). If recruitment is stratified by age but age cohorts stipulated by the PIP opinion do not coincide with the ICH E11 age groups, planned sample sizes will be extracted assuming recruitment will be evenly spread across each cohort that is stipulated by the opinion. Therefore, a study planning to recruit 6 children aged between [7, 13) years and 20 between [14, 18) years, will be recorded as planning to recruit 5 children in the ICH E11 [2-11] years category and 21 in the [12, 18) years category. For the purposes of this analysis, if the total trial sample size exceeds the sum of the sample sizes planned in each age cohort, the sample size at the applicant’s discretion will be imputed as being recruited from the oldest applicable ICH E11 age group concerned by the study. Sample sizes planned by extrapolation based exercises will be imputed as 0 in every ICH E11 age group concerned by the study.

A similar analysis will be conducted to compare the numbers of patients and numbers of PK samples/patient planned across PK trials included in programmes in the Dose-Investigation Set. This analysis will be based upon programmes in the Dose-Investigation Set that include one or more trial measuring PK endpoints, stratifying recruitment (where necessary) and stipulating a PK sampling schedule. The association between the ICH E11 age groups and the number of PK samples/patient will be investigated.

Results for both analyses will be broken down according to the medicine’s orphan status.

**Question 8: What sources of external data are used to determine starting doses in dose-investigation studies that are clinical trials? Are choices supported using M&S?**

**Using DE Q17, All Clinical Trials**. Classify the following as using M&S to determine starting dose choices: a) studies which use simulations based on a PK model to identify a suitable dosing rule and administration schedule for children in the trial. This approach may involve extrapolating the adult PK model and, in particular, adult relationships between demographic covariates and PK parameters, to children. Otherwise, adult PK model parameters may be extrapolated to children by adjusting for demographic covariates using allometric scaling principles; b) studies which use data from previous trials to inform starting dose choices, when at least one of these previous trials is to be analysed using M&S techniques; c) studies which determine starting dose choices using PBPK models developed with adult data and scaled to children.

**Question 9: What steps are taken to validate extrapolation assumptions?**

**Using DE Q21, Dose-Investigation Set:** Calculate the percentage of programmes which plan trials labelled as investigating efficacy in the “study design features” section of the trial synopsis listed in the PIP opinion. Break this percentage down by the number of programmes planning extrapolation based dose-investigation studies.

**Using DE Q19, Q10-11, and Q20.1, All Clinical Trials:** Calculate the percentage of dose-investigation trials using PK guided dose-selection (identified by DE Q19.3) pre-specifying steps to validate the PK-bridging assumption. What percentage of this subgroup plan to measure a PD or efficacy endpoint (as listed in DE Q10 or Q11) and of these trials, what percentage will be analysed by fitting a PK/PD, exposure/response or PK/response model (that is, trials answering yes to DE Q20.1.5)?

# Methodological strengths and weaknesses of dose-investigation studies that are clinical trials.

**Question 10: How robust are the conclusions of dose-investigation studies that are clinical trials? How many dosing rules are compared by dose-ranging clinical trials?**

**Using DE Q12.2 – 12.3, All Clinical Trials & Dose-Varying Clinical Trials:** Calculate the percentage of dose-investigation clinical trials planning to compare more than 1 dosing rule of the novel medicine. For the set of Dose-Varying Clinical Trials, calculate the average number of dosing-rules compared by fixed dose-ranging trials and the percentage of these trials controlled by placebo (or no experimental treatment if the novel medicine is intended for use as an ‘add on’ to standard care).

**Question 11: Is the appropriateness of different dose-investigation study designs related to the age of the study population?**

**Using DE Q12.1, Q13.1, Q14.1, Q15.1 and Q16.1, All Studies:** Summarise the age range for each dose-investigation study by its median. Compare the median (IQR) of these median inclusion ages across the types of study design identified by Question 5.

**Question 12: How will M&S be used to facilitate the design and conduct of dose-escalation trials?**

**Using DE Q14.6 - 14.8, All Clinical Trials:** Compare the percentage of dose-escalation trials specifying model-based vs algorithmic rules for escalation go/no-go decisions. For trials specifying algorithmic rules, give the percentage planning to use rules based on safety or pharmacological information (DE Q14.6.2 – 14.6.3 vs Q14.6.5 --14.6.6). To investigate what criteria are to be used to determine subsequent doses when escalation is permitted, categorise dose increment rules according to the criteria stated in DE Q14.7. Calculate the percentage of dose-escalation trials using on-line M&S to verify/determine dose increments.

**Question 13: How will M&S be used to modify starting doses for staggered cohorts in dose-investigation studies that are clinical trials?**

**Using DE Q9, Q12.6, All Clinical Trials:** Calculate the percentage of fixed dose-strategy trials planning to stagger recruitment into ‘vulnerable’ subgroups. Summarise the demographic variables used to define subgroups as vulnerable. Within the subset of dose-investigation clinical trials staggering recruitment, calculate the percentage planning to use accumulating trial data to modify dosing rules for successive cohorts, the percentage that will use PD/response or PK data to guide modifications, and the percentage that will use M&S to guide dose modifications (and the types of models used for this purpose). Calculate the number of fixed dose-strategy trials planning to use accumulating data to modify individual subjects’ doses in-stream (DE Q12.5).

**Question 14: What analyses are planned for completed dose-investigation clinical trials?**

**Using DE Q20, All Clinical Trials:** Calculate the percentage of trials recorded in each category defined in DE Q20.1.2 – 20.1.9, combining answers to Q20.1.2 and Q20.1.8 as ‘descriptive analyses including summary statistics (PK and PD parameters) and graphics’. Denominator for percentages is total number of dose-investigation studies that are clinical trials planning to measure one or more PK, PD or efficacy endpoint. Calculate the percentage of trials planning to use external data to support model fitting. The denominator for percentages is the total number of trials intending to fit any model (this could include studies with a method of analysis classified as ‘Other’ if the planned analysis will involve fitting a model not captured by DE Q20.1.3 – 20.1.7).

**Question 15: How often are Bayesian methods cited to support the design, conduct or analysis of a dose-investigation study?**

**Using DE Q18, All Studies:** Denominator for percentages is the total number of dose-investigation studies.

**Appendix D:** Data extraction form

1. **PIP application number**
2. **Orphan drug designation inside the EU at time of submission?**
3. **Therapeutic Area of drug**
4. **Article of Paediatric Regulation (1901/2006) the PIP was submitted under (tick appropriate):**

Article 7 (including former Article 30 applications):

Article 8:

1. **Is the indication to be developed only in children or in both adults and children?**

Child only:

Not child-only:

1. **List the lower bound of age range concerned by paediatric development:**
2. **List upper bound of the age range concerned by development (inclusive):**
3. **Will studies be conducted in children to inform future dose selection?**

**If so, answer the following questions:**

**Question 9 relates to the level of experimentation used to support dose-selection in children.**

1. **Is a stepwise approach to testing in more vulnerable subgroups proposed?**

**9.1** State the criteria by which subgroups are defined:

**9.2** State categories defining patient subgroups:

**9.3** Number of responses/time separating enrolment of groups?

**Questions 10 - 11 identify the information upon which dose investigation studies will be based:**

1. **Classify the type of primary endpoint(s) to be measured by the dose-investigation study:**

**10.1** Efficacy (including immunogenicity, (anti-viral) activity, exploratory endpoints)

**10.2** Pharmacokinetic

**10.3** Pharmacodynamic

**10.4** Safety variables (including AEs, tolerability, MTD)

**10.5** Other

1. **Classify the type of secondary endpoint(s) to be measured by the dose- investigation study:**

**11.1** Efficacy (including immunogenicity, (anti-viral) activity, exploratory endpoints)

**11.2** Pharmacokinetics

**11.3** Pharmacodynamic

**11.4** Safety variables (including AEs, tolerability, MTD)

**11.5** Other

**Questions 12 – 16 relate to the design of study(s) to be used to inform the selection of dose(s) to be taken forward for use in future comparative studies or wider clinical practice. More than one type of study may be conducted.**

1. **Will a fixed dose-strategy study be conducted?**

**If so, answer the following questions:**

**12.1** State the age range the study is to be conducted in:

**12.2** State the number of dosing rules to be compared:

**12.3** Is the trial to be placebo controlled?

**12.4 State the total sample size to be evaluated (and by age group):**

**12.4.1** Preterm and term newborns (0 - 27 days inclusive)

**12.4.2** Infants and toddlers (28 days - 23 months inclusive)

**12.4.3** Young children (2 – 11 years inclusive)

**12.4.4** Adolescents (12 – 17 years inclusive)

**12.4.5** Sample size not stratified by age group

**12.5** **Can individual subjects’ doses be modified mid-trial?**

**If yes, state the criteria for modification (if given):**

**12.5.1** Efficacy or PD guided

**12.5.2** PK guided

**12.5.3** Not stated

**12.6** If recruitment is staggered, can starting doses for later cohorts be modified based on accumulating data?

**If yes, are starting dose modifications guided by (assuming that, in addition, modifications would always be informed by safety considerations):**

**12.6.1** Efficacy or PD data

**12.6.2** PK data

**12.6.3** Modelling and/or simulation

1. **Will a therapeutic dose-monitoring trial be conducted?**

**If so, answer the following questions:**

**13.1** State the age range the study is to be conducted in:

**13.2** Will dose-adjustments be based on a target PD/activity response?

**13.3** Will dose-adjustments be based on a target PK response?

**13.4 State the total sample size to be evaluated (and by age group):**

**13.4.1** Preterm and term newborns (0 - 27 days inclusive)

**13.4.2** Infants and toddlers (28 days - 23 months inclusive)

**13.4.3** Young children (2 – 11 years inclusive)

**13.4.4** Adolescents (12 – 17 years inclusive)

**13.4.5** Sample size not stratified by age group

1. **Will a dose escalation study be conducted?**

**If so, answer the following questions:**

**14.1** State the age range the study is to be conducted in:

**14.2 Classify the type of study:**

**14.2.1** Within subject dose-escalation trial

**14.2.2** Sequential cohort, dose-escalation trial

**14.3** State maximum number of (de)-escalations possible in each subject:

**14.4** **State the total sample size to be evaluated (and by age group):**

**14.4.1** Preterm and term newborns (0 - 27 days inclusive)

**14.4.2** Infants and toddlers (28 days - 23 months inclusive)

**14.4.3** Young children (2 – 11 years inclusive)

**14.4.4** Adolescents (12 – 17 years inclusive)

**14.4.5** Sample size not stratified by age group

**14.5** If yes to 14.2.2, how many dosing cohorts will be enrolled?

**14.6** **How will dose escalation decisions be guided?**

**Select the approach used to make dose-escalation go/no go decisions:**

**14.6.1** Forced dose titration (e.g., forced escalation through a set of doses)

**14.6.2** Algorithmic rule based on safety (e.g., 3+3, biased coin, rolling 6)

**14.6.3** Algorithmic rule: pharmacologically (PK or PD) guided dose escalation (eg, escalate until PK or PD target response is attained)

**14.6.4** Model-based rule (e.g., CRM, Modified CRM, EWOC, time-to-event CRM, ffTox, TriCRM, Bayesian logistic regression)

**14.6.5** Unspecified safety analysis

**14.6.6** Unspecified PK analysis

**14.6.7** Not stated

**14.7 Select the rule for determining dose increments:**

**14.7.1** Aiming for target activity level (determined by efficacy/PD/toxicity)

**14.7.2** Aiming for target pharmacological exposure

**14.7.3** Aim to reduce model uncertainty (e.g., D or A optimality)

**14.7.4** Algorithmic rule (e.g. jump to next dose in set/fixed % increase)

**14.7.5** Not known

**14.8** Are dose increments determined/verified by on-line modelling and/simulation?

(If not specified in PIP opinion or application, assume the answer is ‘No’)

1. **Will a cross-over trial be conducted to inform dose-selection?**

**If so, answer the following questions:**

**15.1** State the age range the study is to be conducted in:

**15.2** State the number of (experimental) dosing rules to be compared:

**15.3** Is the trial to be controlled?

**15.4 State the total sample size to be evaluated (and by age group):**

**15.4.1** Preterm and term newborns (0 - 27 days inclusive)

**15.4.2** Infants and toddlers (28 days - 23 months inclusive)

**15.4.3** Young children (2 – 11 years inclusive)

**15.4.4** Adolescents (12 – 17 years inclusive)

**15.4.5** Sample size not stratified by age group

1. **Non-interventional studies informing dose-selection will be conducted.**

**16.1** State the age range the study concerns:

**Select the study design:**

**16.2** M&/S PK/PD/efficacy studies extrapolating from external information

**16.3** Prospective literature review

**16.4** Listing in the PIP opinion completed DI trials conducted in adults and/or

children treated with the current drug (or using such studies as the basis for an

extrapolation study that is listed in the PIP opinion).

**16.5** Listing in the PIP opinion completed DI trials conducted in adults and/children treated with a related product (or using such studies as the basis for an

extrapolation study that is listed in the PIP opinion).

**The following questions relate to how starting doses in the dose-investigation studies will be defined:**

1. **How will starting doses be chosen for dose-investigation trials (in addition to preclinical data)?**

**17.1 Select from the following options:**

**17.1.1** Extrapolating from adult data

**17.1.2** Extrapolating from data in older children

**17.1.3** Extrapolating from children in a related condition (treated with drug of interest)

**17.1.4** Extrapolating from children treated with different formulation of novel drug

**17.1.5** Based on data in correct age group, condition, treated with relevant formulation

of drug of interest

**17.1.6** Other

**17.2**  **Are M&/S techniques cited to support starting dose choices?**

(Including trials which extrapolate relationships between PK model parameters & covariates to children & simulate to find the right dose; modify PK parameters in adult models to adjust for covariates using allometric scaling; determine dose based on data from previous trials which are analysed using modelling techniques).

1. **Are Bayesian methods cited:**

**18.1** To support the design of the dose-investigation study?

**18.2** To support the conduct of the dose-investigation study?

**18.3** For the analysis of the dose-investigation study?

**After dose-investigation studies have been conducted:**

1. **What is the criterion for selecting dose(s) to be used in future comparative trials/clinical use?**

**Select from the following options:** (assuming safety concerns are implicit in all criteria).

**19.1** Maximum tolerated dose

**19.2** Optimum biological dose (e.g. selection guided by PD and/clinical responses)

**19.3** PK guided dose selection

**19.4** Other

**19.5** Not stated

1. **This question concerns how data generated by the dose-investigation trial will be analysed.**

**20.1 How will PK/PD/efficacy data generated by the dose-investigation trials in Q12 - 15 be summarised to support future dose recommendations?**

**20.1.1** Not applicable (only non-interventional studies conducted)

**20.1.2** PK parameters/non compartmental analysis (e.g., Cmin, AUC, t1/2)

(if PK parameters are derived and summarised descriptively, tick boxes 2+8)

**20.1.3** PK model (standard or population PK model)

**20.1.4** PBPK model

**20.1.5** PK/PD, exposure/response or PK/response model

**20.1.6** Dose-exposure model (e.g., log(dose) vs log(AUC) assess dose-proportionality)

**20.1.7** Dose/response, dose/PD model (e.g., ANCOVA); dose-PK-PD models

**20.1.8** Descriptive analyses (e.g., summary statistics including confidence intervals, graphics, summaries of ‘PD parameters’ -- even if it is not clear if these parameters relate to a model).

**20.1.9** Other (other model types fitted/formal hypothesis testing for efficacy endpoints/non-parametric survival analysis)

**20.2 If models are to be fitted, will external data be used to support model fitting?**

(Either trial data combined with data from adults or older children; data pooled across different paediatric studies (possibly different indication). If trial conducted in adults and children and data from both populations analysed together, interpret as ‘Yes’. If not stated, interpret as a ‘No’).

1. **Will efficacy trials be conducted in all ages concerned by medicinal development?**

(Trials described as investigating efficacy/activity/immunogenicity in the “Study design features” section of study synopsis listed in the PIP opinion.)
